# Pain interference, gambling problem severity, and psychiatric disorders among a nationally representative sample of adults

**DOI:** 10.1556/JBA.2.2013.010

**Published:** 2013-06-14

**Authors:** Declan T. Barry, Corey E. Pilver, Rani A. Hoff, Marc N. Potenza

**Affiliations:** ^1^Department of Psychiatry, Yale University School of Medicine, New Haven, CT, USA; ^2^Department of Biostatistics, Yale University School of Medicine, New Haven, CT, USA; ^3^School of Public Health, Yale University School of Medicine, New Haven, CT, USA; ^4^Department of Neurobiology, Yale University School of Medicine, New Haven, CT, USA; ^5^Child Study Center, Yale University School of Medicine, New Haven, CT, USA; ^6^Women's Health Research at Yale, Yale University School of Medicine, New Haven, CT, USA

**Keywords:** pain, mental disorders, comorbidity, gambling

## Abstract

*Background and aims:* A paucity of studies has examined the association between gambling and pain interference. We examined differences in the associations of gambling problem severity and psychiatric disorders among a nationally representative sample of adults with varying levels of pain interference. *Methods:* Chi-square tests and logistic regression analyses were performed on National Epidemiologic Survey on Alcohol and Related Conditions data from 41,987 adult respondents (48% men; 52% women), who were categorized according to two levels of pain interference (i.e., no or low pain interference [NLPI] or moderate or severe pain interference [MSPI]) and three levels of gambling problem severity (i.e., non-gamblers or low-frequency gamblers [NG], low-risk or at-risk gamblers [LRG], and problem or pathological gamblers [PPG]). *Results:* MSPI respondents exhibited higher rates of PPG than NLPI respondents. Categories of Axis I disorders and clusters of mood, anxiety and substance-use disorders showed similarly strong associations with problem-gambling severity in MSPI and NLPI groups. Similarly strong associations between Axis II disorders (and each cluster – A, B and C) and problem-gambling severity were also observed in MSPI and NLPI groups. Exploratory analyses suggested potentially stronger relationships between PPG and dysthymia, panic disorder, and dependent personality disorder and LRG and specific phobia in NLPI compared to MSPI respondents. *Discussion and conclusions:* While MSPI is associated with PPG, largely similar patterns of associations across pain-interference levels were observed between problem-gambling severity and Axis I and Axis II psychiatric disorders.

## Introduction

Pain interference refers to the perceived disruption in daily activities, life roles, interpersonal relationships, and employment resulting from physical pain and is an important outcome variable in the treatment of assorted pain-related medical conditions (Kalliomäki, Meyerson, Gunnarsson, Gordh & Sandblom, 2008; Putzke, Richards, Hicken & DeVivo, 2002). Despite research findings documenting that higher levels of pain interference are associated with increased levels of psychopathology, elevated rates of substance use, and attenuated response among patients to psychiatric treatment, pain interference remains an understudied topic in psychiatric research and residency training (Bair et al., 2004; Barry, Pilver, Potenza & Desai, 2012; Elman, Zubieta & Borsook, 2011; Goldstein, Houck & Karp, 2009).

The extent to which pain interference is associated with gambling problem severity and influences relationships with co-occurring psychiatric disorders – to our knowledge – has not yet been systematically examined. The importance of this subject is suggested from the following lines of research. First, studies indicate that increased gambling problem severity is associated with higher levels of psychiatric and physical health problems and decreased physical functioning – conditions frequently associated with elevated pain interference (Morasco, Pietrzak et al., 2006; Morasco, Vom Eigen & Petry, 2006). Second, chronic pain (i.e., pain lasting at least three months) and pathological gambling have been found to be associated with similar deficits in emotional decision making processes (Apkarian et al., 2004; Goudriaan, Oosterlaan, de Beurs & van den Brink, 2005). Third, individuals with elevated pain interference or pathological gambling may be prone to sedentary lifestyles (Black, Moyer & Schlosser, 2003; Verbunt et al., 2003).

Prior research suggests that elevated pain interference and pathological gambling are each associated with higher levels of psychopathology (Barry et al., 2012; Goldstein et al., 2009; Novak, Herman-Stahl, Flannery & Zimmerman, 2009; Petry, Stinson & Grant, 2005). While published studies on the National Epidemiologic Survey on Alcohol and Related Conditions (NESARC) have examined (a) the prevalence of substance use disorders and their association with general medical conditions based on clinical presentations (e.g., bipolar disorder, gambling disorder), medical issues (e.g., adiposity), or demographic characteristics (e.g., older adults), and (b) the association between pain interference and non-medical use of prescription opioids or a prescription opioid use disorder among the general population and among male and female respondents, separately, they have not – to our knowledge – investigated the prevalence of general medical conditions or substance use among those with varying levels of pain interference based on gambling problem severity (Barry et al., 2012; Goldstein et al., 2009; Goldstein, Dawson, Chou et al., 2008; Goldstein, Dawson, Stinson et al., 2008; Morasco, Pietrzak et al., 2006; Novak et al., 2009; Pietrzak, Morasco, Blanco, Grant & Petry, 2007).

We sought to examine the relationships between socio-demographic characteristics and psychiatric disorders across varying levels of past-year gambling problem severity among respondents with NLPI and MSPI. We hypothesized that the rates of psychiatric disorders would be associated with past-year gambling problem severity in NLPI and MSPI respondents, but the relationship would be weaker in MSPI respondents as compared to NLPI ones given that some of the variance in the relationship between problem-gambling severity and psychiatric disorders would be accounted for by greater pain interference.

## Methods

### Sample

The NESARC was conducted by the US Census Bureau for the National Institute on Alcohol Abuse and Alcoholism and recruited a nationally-representative sample of US non-institutionalized residents (citizens and non-citizens) aged 18 years and older (Grant, Dawson et al., 2003; Grant et al., 2004). The NESARC intentionally over-sampled individuals 18 to 24 years and African American and Hispanic households to provide sufficient statistical power to examine patterns of alcohol use in young people and minority populations. Multi-stage cluster sampling was used to identify respondents: Census sampling units, households, and household members were sequentially sampled. Weights have been computed to adjust standard errors for these over-samples, the cluster sampling strategy, and non-responses (Grant, Moore, Shepard & Kaplan, 2003).

The final NESARC sample in wave one included 43,093 respondents with an overall response rate of 81 percent. For the purposes of this study, we restricted the sample to 41,897 respondents who provided information about their levels of pain interference and gambling problem severity. All participants provided informed consent. The current study of de-identified, publicly accessible data from the NESARC was presented to the Yale Human Investigations Committee, and it was exempted from IRB review under federal regulation 45 CFR Part 46.101(b).

### Measures

*Sociodemographics.* Participants provided information about their gender (male, female), race or ethnicity (White, Black, Hispanic, other), marital status (married, previously married, never married), education (less than high school, high school graduate, some college, college or higher), employment (full time, part time, not working), age, and annual household income.

*Psychiatric disorders.* Trained lay interviewers conducted the Alcohol Use Disorder and Associated Disability Interview Schedule-DSM-IV version (AUDADIS-IV) to collect data related to specific DSM-IV Axis I and Axis II psychiatric disorders (American Psychiatric Association, 1994; Grant, Dawson et al., 2003). The following DSM-IV-related Axis I and Axis II diagnostic variables (derived from AUDADIS-IV) were used in the current study and – consistent with prior research (Grant, Desai & Potenza, 2009) – were grouped as follows: mood disorders (major depression, dysthymia, mania, hypomania); anxiety disorders (panic disorder with or without agoraphobia, social phobia, specific phobia, generalized anxiety disorder); substance use disorders (alcohol abuse/dependence, drug abuse/dependence, nicotine dependence); and personality disorders belonging to Clusters A (paranoid, schizoid), B (histrionic, antisocial), and C (avoidant, dependent, obsessive–compulsive). Interviewers assessed general medical condition and substance use exclusions related to past-year Axis I diagnoses; thus, research diagnoses can be viewed as “primary” as per DSM-IV/DSM-IV-TR guidelines (American Psychiatric Association, 1994; Desai & Potenza, 2008).

*Pain interference.* Pain interference was examined using a subscale from the 12-Item Short-Form Health Survey (SF-12) (Ware, Kosinski & Keller, 1996). Similar to previous research, respondents' answers to the 5-point item: “During the past 4 weeks, how much did pain interfere with your normal work (including both work outside the home and housework)” were used to classify them as having either: a) “no or low pain interference” (i.e., those reporting their pain interference as “not at all” or “a little bit”) or b) “moderate or severe pain interference” (i.e., those reporting their pain interference as “moderate”, “a lot” or “extreme”) (Thomas, Peat, Harris, Wilkie & Croft, 2004).

*Problem-gambling severity.* Similar to prior studies, respondents' answers to the gambling-related items from the AUDADIS-IV were used to classify them into one of three gambling groups: a) “non-gamblers or low-frequency gamblers” (i.e., those reporting that they had never gambled more than five times per year in their lifetime); b) “low-risk or at-risk gamblers” (i.e., those reporting gambling more than five times in a year but who exhibited 0 to 2 inclusionary criteria of pathological gambling in the previous year); and c) “problem or pathological gamblers” (i.e., those reporting 3 or more inclusionary criteria of pathological gambling in the previous year) (Desai & Potenza, 2008).

### Data analysis

The primary research questions addressed whether there were differences in the association between past-year gambling problem severity and psychiatric disorders among respondents reporting NLPI, compared to those with MSPI. First, using chi-square tests (c^2^), we examined the associations between gambling problem severity and sociodemo-graphic characteristics (race/ethnicity, marital status, education level, employment status, age, and household annual income), stratified by pain interference levels (NLPI and MSPI), in order to identify sociodemographic variables potentially influencing the relationship between pain interference levels, gambling problem severity, and psychiatric disorders. Second, we examined the unadjusted weighted prevalence of psychiatric disorders, stratified by both pain interference levels and gambling problem severity. Third, we fit a series of binary logistic regression models with psychiatric variables as the dependent variable of interest and the 3-level gambling problem severity variable (i.e., non-gamblers or low-frequency gamblers [NG], low-risk or at-risk gamblers [LRG], problem or pathological gamblers [PPG]), 2-level pain interference (NLPI and MSPI), and the interaction between gambling problem severity and pain interference level as the independent variables of interest, adjusting for potentially confounding sociodemographic variables(i.e., race/ethnicity, marital status, education, age, employment, household annual income). Our analysis began by examining psychiatric disorders grouped into Axis I and Axis II categories within each pain-interference group. If significant findings were observed, 3 categories within each Axis were examined within each pain-interference group to investigate further the nature of the findings: any mood disorder, any anxiety disorder, and any substance use disorder for Axis I categories, and any Cluster A, any Cluster B, and any Cluster C for Axis II categories. When significant associations were found between these categories and pain interference levels and gambling problem severity, we pursued further analysis of the individual disorders within each pain-interference group. The NG category was used as a reference level for two sets of adjusted odds ratios: LRG versus NG and PPG versus NG. Interaction term odds ratios tested whether the adjusted odds ratios for no or low pain interference respondents were significantly different from those for moderate or severe pain interference respondents. Given the complex design of the study sample and the goal of estimating as accurately as possible the national rates of co-occurring psychiatric disorders, analyses were performed using NESARC-calculated weights and SUDAAN software (Re-search Triangle Institute, 2001). Consequently, sample proportions are based on weighted percentages.

## Results

### Sociodemographics

A plurality of respondents were women (52.2%), were married (62.0%), self-identified as white (71%), had at least a high school level of education (84%), were working full-time (53.3%), and reported an annual household income exceeding $34,999 (58.0%) (weighted percentages provided). The patterns of associations between gambling problem severity and sociodemographic characteristics were largely similar across respondents with NLPI and MSPI; generally, the magnitude of the associations between gambling problem severity and sociodemographics were numerically higher among those with NLPI compared to those with MSPI (Table 1).

### Problem-gambling severity

Problem-gambling severity was significantly associated with level of pain interference in bivariate analysis (*p* =0.0098). The majority of respondents irrespective of their level of pain interference reported either NG (72.5% and70.8% for respondents with NLPI and MSPI, respectively) or LRG (27.1% and 28.4% for respondents with NLPI and MSPI, respectively). The prevalence of PPG was higher for MSPI (0.79%) respondents in comparison to NLPI (0.48%) respondents (*p* = 0.0182). Overall, 0.54% of the total sample reported PPG.

**Table 1.** Sociodemographic characteristics of no/low and moderate/severe pain interference respondents by gambling problem severity^1^

Characteristics	No/low pain respondents					Moderate/severe pain respondents				
	NG	LRG	PPG			NG	LRG	PPG		
	*n* = 24,542^2^	*n* = 8,522^2^	*n* = 165^2^	χ^2^	*P*	*n* = 6,224^2^	*n* = 2,376^2^	*n* =68^2^	χ^2^	*P*
	%	%	%			%	%	%		
Gender				52.83	< 0.001				31.97	< 0.001
Male	44.9	60.4	67.7			38.0	51.1	57.6		
Female	55.1	39.6	32.3			62.0	48.9	42.4		
Race/ethnicity				7.07	< 0.001				4.33	0.001
White	69.1	75.6	56.7			70.3	75.1	67.8		
Black	10.9	10.25	21.0			11.8	11.7	23.7		
Hispanic	13.3	8.3	8.1			11.5	7.2	3.3		
Other	6.7	5.9	14.3			6.4	6.0	5.2		
Marital status				13.92	< 0.001				3.80	0.008
Married	61.8	65.4	39.8			58.5	61.1	65.2		
Previously married	14.8	15.9	22.5			26.6	27.0	12.5		
Never married	23.4	18.8	37.6			14.9	11.9	22.3		
Education				8.93	< 0.001				2.70	0.021
Less than HS	14.1	11.2	18.2			26.1	22.1	22.5		
HS graduate	27.9	30.6	35.11			31.6	32.2	43.3		
Some college	30.0	32.8	29.2			26.7	31.5	24.2		
College or higher	28.1	25.4	17.5			15.7	14.2	10.0		
Employment				6.33	< 0.001				3.80	0.008
Full time	57.4	61.9	62.5				30.8	35.8	39.0	
Part time	11.4	10.1	12.2				8.5	7.7	13.2	
Not working	31.3	28.0	25.3				60.8	56.5	47.8	
Age (mean age ± *SD*)^3^	42.6	45.6	40.4	65.6^4^	< 0.001	52.5	53.1	42.1	10.34^4^	< 0.001
Income				12.39	< 0.001				7.00	< 0.001
$0-19,999	19.8	14.2	21.3			36.4	27.6	34.4		
$20,000-34,999	19.7	18.9	20.7			21.8	22.2	23.2		
$35,000-69,999	33.7	36.9	35.4			27.3	31.1	25.7		
$70,000+	26.8	30.0	22.6			14.5	19.1	16.7		

*Notes:* NG = no gambling or low-frequency gambling group; LRG = low-risk or at-risk gambling group; PPG = problem or pathological gambling group; HS = high school. ^1^ Proportions in table represent weighted percentages, stratified by pain interference. ^2^ Ns represent actual number in each category. ^3^ Numbers represent weighted mean values, stratified by pain interference. ^4^ F test.

### Psychiatric disorders

Table 2 summarizes the patterns of associations observed between problem-gambling severity and psychiatric morbidity stratified by level of pain interference (i.e., NLPI vs. severity within each pain-interference group was related to each Axis category and the groups within each Axis, the strengths of the associations between problem-gambling severity and these Axis I and Axis II groups were similar across the pain-interference groups. Several individual disorders showed differences across pain-interference groups in the strengths of the associations with problem-gambling severity. The relationships between PPG and dysthymia MSPI). Within each pain-interference group, significant associations between problem-gambling severity were observed for any Axis I and any Axis II disorder for respondents with NLPI and and for those with MSPI, and significant associations were also found within each contributing category in Axis I (any mood disorder, any anxiety disorder, and any substance-use disorder) and Axis II (any Cluster A personality disorder, any Cluster B personality disorder, and any Cluster C personality disorder) disorder domains in both pain interference groups.

**Table 2.** Prevalence of psychiatric diagnoses by gambling problem severity among respondents with no/low and moderate/severe pain interference^1^

Psychiatric diagnoses	No/low pain respondents					Moderate/severe pain respondents				
	NG	LRG	PPG			NG	LRG	PPG		
	*n* = 24,542^2^	*n* = 8,522^2^	*n* = 165^2^	χ^2^	*P*	*n* = 6,224^2^	*n* = 2,376^2^	*n* =68^2^	χ^2^	*P*
	%	%	%			%	%	%		
Any Axis I disorder	25.8	37.2	72.1	56.82	< 0.001	34.3	46.2	78.0	27.18	< 0.001
*Any mood disorder*	8.6	9.3	27.5	9.98	< 0.001	14.4	17.8	26.3	5.60	0.006
Major depression	6.2	5.6	16.2	5.11	0.009	11.2	13.1	13.9	1.37	0.261
Dysthymia	1.3	1.3	7.1	2.60	0.082	3.9	4.3	2.7	0.58	0.564
Mania	1.2	1.6	8.8	5.98	0.004	2.6	4.2	7.9	5.13	0.009
Hypomania	1.8	2.8	6.5	8.41	< 0.001	1.7	2.4	9.0	2.77	0.070
*Any anxiety disorder*	9.2	11.9	27.9	17.19	< 0.001	16.0	19.6	34.6	7.37	0.001
Panic disorder w/ or w/o agoraphobia	1.5	1.8	8.7	2.80	0.068	4.1	5.4	3.3	1.79	0.176
Social phobia	2.3	2.8	9.7	3.61	0.033	4.0	4.9	9.3	2.18	0.121
Specific phobia	6.0	8.2	21.2	17.72	< 0.001	9.5	11.3	21.7	3.28	0.044
Generalized anxiety disorder	1.5	1.5	7.9	1.97	0.147	4.0	5.9	7.2	3.86	0.026
*Any substance use disorder*	15.2	25.9	55.9	60.77	< 0.001	18.0	30.3	60.4	30.16	< 0.001
Alcohol abuse/dependence	7.0	13.0	31.3	43.62	< 0.001	5.6	10.8	29.0	17.21	< 0.001
Drug abuse/dependence	1.6	2.9	5.9	11.27	< 0.001	1.9	3.1	5.9	3.03	0.055
Nicotine dependence	9.7	17.0	43.1	52.90	< 0.001	14.6	23.3	44.2	22.13	< 0.001
Any Axis II disorder	12.2	17.4	53.2	35.99	< 0.001	18.2	26.3	56.0	21.23	< 0.001
*Any Cluster A*	4.9	6.3	25.6	15.12	< 0.001	9.6	12.4	34.0	8.03	< 0.001
Paranoid	3.4	4.4	22.8	12.32	< 0.001	6.9	8.9	33.5	7.46	0.001
Schizoid	2.4	3.1	13.3	8.55	< 0.001	5.0	6.8	21.9	5.40	0.007
*Any Cluster B*	3.7	6.7	28.2	26.98	< 0.001	5.7	10.1	28.2	17.16	< 0.001
Histrionic	1.5	1.9	12.8	7.59	0.001	2.5	3.6	14.8	4.35	0.017
Antisocial	2.6	5.2	18.8	23.88	< 0.001	3.9	7.6	25.4	16.48	< 0.001
*Any Cluster C*	7.8	10.4	27.0	16.14	< 0.001	12.0	16.5	34.1	11.96	< 0.001
Avoidant	2.0	1.7	9.6	3.99	0.023	4.0	4.6	9.8	1.76	0.181
Dependent	0.3	0.3	3.0	1.96	0.149	1.4	1.2	2.5	0.67	0.513
Obsessive-compulsive	6.6	9.3	22.0	19.08	< 0.001	9.6	13.7	31.1	11.31	< 0.001

*Notes:* NG = no gambling or low-frequency gambling group; LRG = low-risk or at-risk gambling group; PPG = problem or pathological gambling group. ^1^ Proportions in table represent weighted percentages, stratified by pain interference. ^2^ Ns represent actual number in each category.

Adjusted odds ratios from multivariate models investigating the strength of associations between psychiatric disorders and problem-gambling severity groups are presented for respondents with NLPI and MSPI, using NG as the reference group (Table 3). The odds of any Axis I disorder, any anxiety disorder, any substance-use disorder, any Axis II disorder, any Cluster A personality disorder, any Cluster B personality disorder, and any Cluster C personality disorder were elevated for LRG and PPG (in comparison to NG) in both NLPI and MSPI respondents. While problem-gambling (OR = 8.74, *p* < 0.01), panic disorder with or without agoraphobia (OR = 9.86, *p* < 0.05), and dependent personality disorder (OR = 6.38, *p* < 0.05) were stronger among participants with NLPI compared to participants with MSPI. Additionally, the association between LRG and specific phobia (OR = 1.24, *p* < 0.05) was stronger among participants with NLPI compared to participants with MSPI.

## Discussion and Conclusions

This study is the first, to our knowledge, to systematically investigate differences between adults with varying levels of pain interference in the associations between Axis I and Axis II psychiatric disorders and different levels of gambling problem severity in a nationally representative sample. The findings largely did not support our *a priori* hypothesis. The relationship between past-year problem-gambling severity and psychopathology was largely similar across Axis I and Axis II disorders and major groupings of these disorders. Data suggest that the relationships might vary for only four disorders. Specifically, the relationships appeared stronger at *p* < 0.05 among respondents with NLPI compared to those with MSPI between PPG and dysthymia, panic disorder with or without agoraphobia, and dependent personality disorder and between low-risk or at-risk gambling and specific phobia.

**Table 3.** Associations between psychiatric diagnoses and gambling severity among respondents with no/low and moderate/severe pain interference

Psychiatric diagnoses	No/low pain respondents		Moderate/severe respondents		Interac (no/low vs. moderate/severe pain)	
	OR for LRG vs. NG	OR for PPG vs. NG	OR for LRG vs NG	OR for PPG vs. NG	OR for LRG vs. NG	OR for PPG vs. NG
Any Axis I disorder	1.90^***^	7.74^***^	1.73^***^	5.88^***^	1.10	1.32
*Any mood disorder*	1.31^***^	4.20^***^	1.45^***^	1.93	0.90	2.18
Major depression	1.09	3.08^***^	1.35^**^	1.22	0.81	2.53
Dysthymia	1.24	5.89^***^	1.26	0.67	0.99	8.74^**^
Mania	1.60^***^	7.33^***^	1.94^***^	2.39	0.83	3.07
Hypomania	1.76^***^	3.31^**^	1.49	4.22^**^	1.18	0.79
*Any anxiety disorder*	1.58^***^	4.67^***^	1.42^***^	2.84^**^	1.11	1.64
Panic disorder w/ or w/o agoraphobia	1.44^**^	7.59^***^	1.53^**^	0.77	0.95	9.86^*^
Social phobia	1.35^**^	4.96^***^	1.36^*^	2.37	1.00	2.09
Specific phobia	1.67^***^	5.37^***^	1.35^**^	2.66^*^	1.24^*^	2.01
Generalized anxiety disorder	1.28	6.46^***^	1.71^***^	1.82	0.75	3.55
*Any substance use disorder*	2.07^***^	6.73^***^	2.03^***^	5.41^***^	1.02	1.24
Alcohol abuse/dependence	2.06^***^	5.08^***^	2.04^***^	5.01^***^	1.01	1.01
Drug abuse/dependence	2.12^***^	2.64^**^	1.94^**^	1.80	1.09	1.47
Nicotine dependence	1.99^***^	6.62^***^	1.80^***^	3.53^***^	1.11	1.87
Any Axis II disorder	1.63^***^	7.81^***^	1.65^***^	4.81^***^	0.98	1.62
*Any Cluster A*	1.52^***^	6.00^***^	1.46^***^	4.07^***^	1.04	1.48
Paranoid	1.59^***^	7.58^***^	1.50^**^	5.87^***^	1.06	1.29
Schizoid	1.45^***^	5.63^***^	1.49^**^	4.39^***^	0.97	1.28
*Any Cluster B*	1.96^***^	8.25^***^	1.91^***^	4.33^***^	1.02	1.91
Histrionic	1.54^***^	8.19^***^	1.64^**^	4.94^**^	0.94	1.66
Antisocial	2.08^***^	6.64^***^	2.06^***^	5.28^***^	1.01	1.26
*Any Cluster C*	1.43^***^	4.48^***^	1.48^***^	3.38^***^	0.97	1.33
Avoidant	1.00	4.86^***^	1.28	2.20	0.78	2.21
Dependent	1.13	9.81^***^	1.01	1.54	1.12	6.38^*^
Obsessive-compulsive	1.48^***^	4.18^***^	1.49^***^	3.83^***^	0.99	1.09

*Notes:* NG = no gambling or low-frequency gambling group; LRG = low-risk or at-risk gambling group; PPG = problem or pathological gambling group; OR = odds ratio. Adjusted for gender, race/ethnicity, marital status, age, education, employment, and household annual income. ^*^ *p* < 0.05, ^**^ *p* < 0.01, ^***^ *p* < 0.001.

### Pain interference, problem-gambling severity, and psychiatric disorders

Our findings largely corroborate those previously reported on the high rates of co-occurrence between PPG and Axis I and Axis II psychiatric disorders among patients in treatment or seeking help (Barry, Steinberg, Wu & Potenza, 2008; Crockford & El-Guebaly, 1998; Shaffer & Korn, 2002) and among respondents in epidemiological studies (Cunningham-Williams, Cottler, Compton & Spitznagel, 1998; Petry et al., 2005). Study findings confirm and expand upon those reported in prior studies by demonstrating that associations between problem-gambling severity and psychopathology are largely not modified by levels of pain interference. However, we found that pain interference might modify the association between problem-gambling severity and four specific psychiatric disorders: the relationships between PPG and dysthymia, panic disorder with or without agoraphobia, and dependent personality disorder appeared more robust among respondents with NLPI as compared to their counterparts with MSPI, whereas the relationship between low-risk or at-risk gambling and specific phobia appeared stronger among participants with NLPI compared to those with MSPI. These findings suggest that some of the relationship between problem-gambling severity and these disorders may relate importantly to MSPI.

It is currently unclear why pain interference might modify the associations between problem-gambling severity and some specific psychiatric disorders. It is possible, for example, that gambling activities may serve as a distraction from pain and its associated psychopathology. While sustained attention on pain appears to explain in part the disabling effects of persistent pain, the extent to which gamblers who have pain engage in gambling as a distraction strategy has not been systematically examined (Eccleston & Crombez, 2007). The stronger association of LRG and specific phobia among participants with NLPI compared to those with MSPI expands upon prior research that documented the importance of assessing and addressing the correlates of subsyndromal gambling and not only those related to PPG (Barry et al., 2008).

### Limitations and strengths

Several potential limitations are worth noting. The cross-sectional design of the NESARC limits statements regarding causation among study variables. Similar to previous epidemiologic and community studies, pain interference in this study was examined using a single item from the SF-12 (Barry et al., 2012; Goldstein et al., 2009; Novak et al., 2009). Future research in this area might benefit from using a more comprehensive pain interference scale (e.g., Brief Pain Inventory-Short Form [Cleeland, 1991]). Because of concerns about response burden, the NESARC did not exhaustively assess Axis I or Axis II psychiatric diagnoses or general medical conditions; thus, certain diagnoses of potential clinical relevance to levels of pain interference were not assessed, including somatoform disorder and borderline personality disorder.

Despite these limitations, the current study represents an investigation of differences in the psychiatric morbidity accompanying varying levels of problem-gambling severity among respondents with no or low and moderate or severe pain interference. To our knowledge, this study is among the first to systematically examine differences in psychiatric disorders accompanying problem-gambling severity among a nationally representative sample of individuals in the United States with varying levels of past-month pain interference. Higher levels of problem or pathological gambling were found to occur in individuals with MSPI as compared to those with NLPI, and level of pain interference may influence the relationships between problem-gambling severity and several psychiatric disorders. Specifically, findings from interaction analyses suggest that MSPI may account for some of the variance in the associations between PPG and dysthymia, panic disorder, and dependent personality disorder, and between low-risk or at-risk gambling and specific phobia. These findings suggest that treatments for PPG might benefit from assessing and addressing pain interference levels in addition to psychiatric disorders, especially among patients with dysthymia, panic disorder, or dependent personality disorder.
